# Business Notices

**Published:** 1878-06

**Authors:** 


BUSINESS NOTICES.—*Address*—Always give name of County as well as that of Post-office. Removals should be promptly reported, that the address may be changed and loss to the subscriber prevented. *Remittances* should be made by Post-office order, by check or registered letter. Where a check is sent, it should be on a National Bank in New York City, otherwise the collection of it costs 25 cents, which will hereafter be charged against the subscriber. If private checks are sent, 25 cents additional (for collection) should be always added; the aggregate loss to the JOURNAL otherwise is very great. *Receipts* for all money received are sent immediately. No discontinuances will be made unless arrearages are paid; this is a postal law sustained in all courts to prevent serious loss to publishers. Refusal to remove a JOURNAL from the Post-office does not exempt the subscriber from liability for its cost, so long as arrearages are unpaid. When arrearages are paid and a subscriber wishes to have his JOURNAL discontinued, he should so notify the publisher by card or letter. Sending a JOURNAL back to the offiee of publication gives no information as to the name of the sender, unless his hame be written on the package, and this subjects him to a heavy fine. *Duns* are painful to receive, but far more painful to send. They are easily and simply avoided. To complain of them is unreasonable and unjust. Removing from any neighborhood and failing to notify a publisher to stop sending a periodical to that address is regarded by postal laws *prima facie* evidence of fraud, unless the accumulated arrearages be paid on demand. Without the protection of such a law, publishers are exposed to very heavy loss.

